# Number of screws in the articular segment of distal humerus AO/OTA C-type fractures treated with open reduction internal fixation is associated with complication rate

**DOI:** 10.1051/sicotj/2021006

**Published:** 2021-04-01

**Authors:** Brian F. Grogan, Nicholas C. Danford, Cesar D. Lopez, Stephen P. Maier, Pinkawas Kongmalai, David Kovacevic, William N. Levine, Charles M. Jobin

**Affiliations:** 1 Department of Orthopedic Surgery, Columbia University Irving Medical Center 622 W. 168th St. PH-11 New York 10032 NY USA; 2 Department of Orthopedics and Rehabilitation, University of Wisconsin School of Medicine and Public Health, UW Health at The American Center 4602 Eastpark Boulevard Madison 53718 WI USA

**Keywords:** Distal humerus fracture, Open reduction and internal fixation, Elbow, Trauma

## Abstract

*Introduction*: Surgical treatment of distal humerus fractures can lead to numerous complications. Data suggest that the number of screws in the distal (articular) segment may be associated with complication rate. The purpose of this study is to evaluate the association between a number of screws in the distal segment and complication rate for surgical treatment of distal humerus fractures. We hypothesize that the number of screws in the articular segment of distal humerus AO/OTA C-type fractures treated with open reduction internal fixation (ORIF) will be inversely proportional to the complication rate. *Methods*: We performed a single-center retrospective cohort study of 27 patients who underwent ORIF of distal humerus fractures C-type with at least six months of radiographic and clinical follow-up. Clinical outcomes including a range of motion, pain, revision surgery for stiffness and/or heterotopic ossification (HO), nonunion, and persistent ulnar nerve symptoms requiring revision neurolysis were recorded. *Results*: In C-type fractures, the use of three or fewer articular screws was significantly associated with nonunion or loss of fixation (RR 17, *p* = 0.006). Nineteen of 36 (53%) patients experienced at least one complication. The surgical approach, plate configuration, age, and ulnar nerve treatment (none, in situ release, transposition) were not associated with the need for revision surgery. Men had a higher risk of requiring surgical contracture release due to improving post-operative stiffness (RR 12, *p* = 0.02). *Conclusion*: In this retrospective study, the use of three or fewer screws to fix articular fragments in AO type C fractures was a significant risk for nonunion or loss of fixation. Plate configuration and surgical approach did not correlate with outcomes. Men had higher rates of complications and required more frequent revision surgery compared to women.

## Introduction

Fractures of the distal humerus make up 2–6% of all humeral fractures and 30% of all elbow fractures [1]. These fractures can be difficult to treat surgically. Complications of distal humerus open reduction and internal fixation (ORIF) occur in up to 35% of patients [2–4]. They include nonunion, ulnar nerve injury, and stiffness secondary to heterotopic ossification, all of which may require revision surgery. Ulnar neuropathy has been reported to occur in 7–15% of cases, is more common in C-type fractures, and may be related to plate placement [5–10]. Nonunion occurs in approximately 0–7% of cases [11–13] and heterotopic ossification has a varying prevalence in the literature.

The association of these complications with important elements of operative decision making such as plate and/or screw construct is poorly understood, with certain literature suggesting that more screws in the distal articular segment of AO Foundation/Orthopaedic Trauma Association (AO/OTA) C-type fractures may be associated with fewer complications [4]. We, therefore, performed this study with the purpose of evaluating risk factors for complications of distal humerus ORIF. We hypothesized that there would be an association between a number of screws in the articular segment of an AO/OTA C-type fracture and complications, with an association between a decreasing number of screws and an increased complication rate.

## Material and methods

This is a single-center retrospective cohort study of 27 consecutive unselected patients who underwent ORIF of distal humerus fractures AO type C between 2007 and 2017. Inclusion criteria were at least six months of radiographic and clinical follow-up. Patients with open fractures, distal humerus non-union, fewer than six months of follow up, or previous distal humerus surgery were excluded. Patients who received delayed surgical treatment (defined as delay greater than four weeks from presentation) were also excluded. The Institutional Review Board approved this study.

There were 27 patients with C-type distal humerus fractures treated with ORIF who met inclusion criteria. The average follow-up time was 15.9 months. Fourteen patients (52%) were women, and the average age at the time of surgery was 49.9 years (range: 16–87), with almost half (*n* = 13) over the age of 50. Thirteen (48%) patients underwent olecranon osteotomy during surgical exposure, and 23 of 27 (85%) had ulnar nerve transposition or neurolysis at the time of the initial surgery (Table 1). Sixteen (59%) cases included compression of the columns.

**Table 1:** Results.

	Type C fractures
Sample size	27
Average age	49.9
Age SD	18.5
Age range	16–87
Over age 50	13 (48%)
Male	13 (48%)
Female	14 (52%)
Olecranon osteotomy	13 (48%)
Ulnar nerve transposition or neurolysis at index	23 (85%)
Screws in articular fragments	5.2
Locking screws	3.1
Any complication	15 (56%)
Elbow stiffness	9 (33%)
Ulnar nerve symptoms requiring revision	3 (11%)
Nonunion/Malunion	3 (11%)
Infection	1 (4%)
Contracture requiring release	8 (30%)
HO requiring excision	4 (15%)

Procedures were all performed at one institution by six board-certified orthopedic surgeons, all using Acumed Distal Humerus Locking Plate system (Acumed LLC, Hilsboro, OR, USA), Stryker Variax Distal Humeral Locking Plate System (Stryker Orthopedics, Mahwah, NJ, USA), or Synthes Reconstruction Plate System (DePuy Synthes, West Chester, PA, USA), or cannulated headless screws (Acumed or Stryker) and/or K-wires. No fractures were fixed with only headless screws and K-wires.

Patient demographic information was recorded along with the surgical approach, initial management of the ulnar nerve (not dissected, *in-situ* release, anterior submuscular transposition, anterior subcutaneous transposition), AO/OTA fracture pattern including presence or absence of a coronal shear fracture pattern of the capitellum or trochlea, plate configuration, use of distal locking or non-locking screws, and a number of screws in the articular fragments. We defined an articular screw as any screw that held any piece or all of an articular fragment (fragment composed partially of articular cartilage), which is based on previous literature [4]. Post-operative radiographs were evaluated for fracture union, loss of fixation, hardware complications, and heterotopic ossification. Clinical outcomes including a range of motion as measured with a goniometer, pain, revision surgery for stiffness and/or heterotopic ossification (HO), nonunion, other revision surgery, and persistent ulnar nerve symptoms requiring revision neurolysis were recorded. The analysis included descriptive statistics and multivariate regression analysis to identify factors significantly associated with these clinical outcomes. A *P*-value less than 0.05 was considered significant.

## Results

Fifteen (56%) patients experienced at least one complication including: elbow stiffness with less than 30–130 degrees arc of motion (*n* = 9), ulnar nerve symptoms requiring revision surgery (*n* = 3), nonunion or malunion (*n* = 3), infection (*n* = 1), and revision surgery for contracture release (*n* = 8) or HO excision (*n* = 4).

Plate configuration, presence of articular coronal shear fragment, and implant manufacturer did not correlate significantly with complication rate (Table 2). The average number of articular screws used in type C fractures was 5.2, with an average of 3.1 locking screws. Twenty-three (85%) cases had more than three articular screws. The average number of screws through the plate was 4.6, and the average number of screws engaging the contralateral fragment was 3.1. The use of 3 or fewer articular was significantly associated with non-union or loss of fixation (RR 17, *p* = 0.006) (Table 3). Radiographic evidence of articular reduction and complete bony healing in a patient with type C fracture caused by a fall from height is displayed. Among the four non-union cases, three (75%) involved the articular surface, while one non-union case (25%) was diaphyseal. A total of three (75%) non-union cases involved placement of eccentric compression screws on the humeral shaft and all four (100%) had sufficient proximal fixation and inter-fragmentary lag screws placed across the diaphyseal fragment.

**Table 2:** Complication rates by device manufacturer.

Implant manufacturer	Number of ORIF (*n*)	Any complication (*n*)	(%)	Complication excluding stiffness (*n*)	(%)	Stiffness only (*n*)	(%)
Acumed locking	23	13	56%	10	43%	3	13%
Stryker variax	3	2	67%	1	33%	1	33%
Synthes reconstruction	1	0	0%	0	0%	0	0%
Total	27	15	56%	11	41%	8	15%

**Table 3:** Risk of nonunion or loss of fixation in patients with distal humerus C-type fractures treated with ORIF using 3 or fewer articular screws versus 4 or greater articular screws.

# of screws	Nonunion absent	Nonunion present
3 or fewer	1	3
4 or greater	22	1
		RR 17 (*p* = 0.006)

Men were more likely to have any complication compared to women (RR for all complications 6.9, *p* = 0.02; RR for elbow stiffness 32, *p* = 0.005; RR for revision surgery 2.2, *p* = 0.009; RR for HO 18, *p* = 0.04). The majority of elbow motion lost was in flexion, with an average difference of 21° between men and women (*p* = 0.01). Loss of supination was significantly associated with male gender (−8°, *p* = 0.02), age over 50 years (−8°, *p* = 0.02), and an olecranon osteotomy for exposure (−7°, *p* = 0.04). Older patients (>50 years of age) also had greater loss of pronation compared to patients less than 50 years of age (20° less pronation, *p* = 0.03). The need for revision surgery was not associated with the surgical approach, plate configuration, age over 50 years, or ulnar nerve treatment.

## Discussion

**Figure 1:**
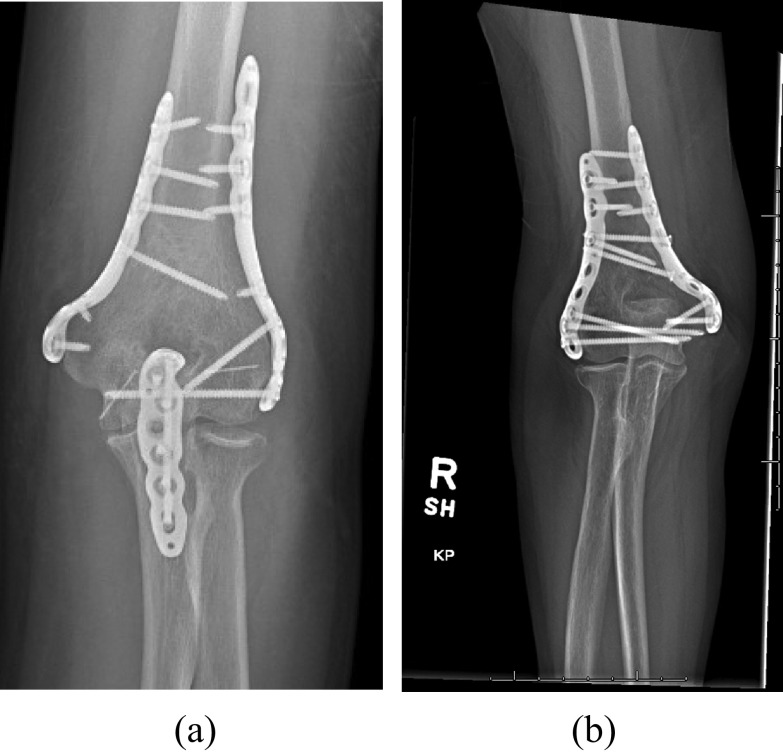
(a) AP radiograph 2 years post-op showing placement of 2 screws in distal fragment. Parallel plating of the distal humerus shaft is also shown in the image. There is osseous bridging across a transfixed olecranon osteotomy. (b) AP radiograph 2 years post-op showing articular reduction and complete bony healing of distal humerus fracture. The repaired construct included parallel plating with 5 articular fragment locking screws with interdigitation between the medial and lateral plate screws.

Surgical treatment of distal humerus fractures can lead to numerous complications, and previous studies have suggested that the number of screws in the distal (articular) segment may be associated with complication rate [4, 6–10, 14, 15]. Our study supports using more than three articular screws in AO/OTA C-type distal humerus fractures, as we found a significant correlation between three or fewer articular screws and complication rate. This finding confirms our hypothesis that a greater number of screws is optimal when obtaining fixation in the articular segment of distal humerus fractures. Other evidence supports the use of more articular screws as well, which may be associated with complete bony healing after ORIF (Figures 1a and 1b) [4, 6, 7, 16]. Plate configuration (perpendicular vs. parallel) was not significantly associated with complication rate. This result is in line with previously published clinical and biomechanical data on plate configuration [16–21]. Additionally, men and patients greater than 50 years of age may be at increased risk of complication of distal humerus ORIF compared to women.

The limitations to our study include a small sample size, including for non-unions of C-type fractures. Other limitations including those inherent in a retrospective cohort review, such as lack of a control group. Furthermore, certain variables that we did not account for may have affected complication rates such as patient bone quality, screw length, and sub-type of fracture. The findings of this analysis are not directly conclusive and do not show direct causation, but instead demonstrate a statistical correlation between certain risk factors and outcomes.

Although the present study did not use computed tomography (CT) imaging to evaluate fractures and bony healing outcomes, some published studies have used CT for analysis of distal humerus fractures [22, 23]. Jacquot et al. reported that CT improves the diagnostic accuracy of adult distal humerus fractures compared to radiographs (95% vs. 73% accuracy, respectively), and in some cases, even influenced the surgical strategy [23]. A multicenter, cross-sectional study in Japan used CT scans to determine differences in distribution and fracture patterns between low- and high-energy distal humerus fractures and determine fracture classification [22]. Thus CT does play a role in diagnostic work-up, fracture classification, and surgical planning. We did not rely on it for clinical follow-up, as plain radiography allowed for assessment of a number of screws used in the articular segment of C-type distal humerus fractures its association with important clinical outcomes.

Although several studies have attributed non-union and other complications to plating, some studies have shown that screws play an important role in the success of the plating type [4, 6–10, 14, 15]. Claessen et al. found a stronger association between poor fixation of the distal fragment and revision for nonunion or implant complications than with plating type [6]. Their study showed that most of the nonunion cases either had an inadequate number of screws or screws that were not long enough that were placed through the plate into the distal fragments. In a report by Jayakumar and Ring, it was suspected that the use of pre-contoured, fixed angle distal humerus plates caused surgeons to use too few distal locking screws, many of which were too short, which resulted in two cases (67%) of axial failure out of three total patients [7]. As a result, they recommended that surgeons place as many screws as possible in the distal fragment and that each screw has sufficient length to pass through the fragments into the distal cortex and the screws should be placed through a plate. O’Driscoll et al. concluded that the best way to optimize stability in distal humerus fracture fixation is to ensure the screws maximize fixation in the distal fragment and stability between the distal fragment and the shaft [4] (Table 4).

**Table 4:** Key attributes to successful fixation (O’Driscoll [4]).

Fixation principles
Fixation in the distal fragment must be maximized. All fixation in distal fragments should contribute to stability between the distal fragments and the shaft.
Fixation objectives
Every screw in the distal fragments should pass through a plate. Engage a fragment on the opposite side that is also fixed to a plate. As many screws as possible should be placed in the distal fragments. Each screw should be as long as possible. Each screw should engage as many articular fragments as possible. The screws in the distal fragments should lock together by interdigitation, creating a fixed-angle structure. Plates should be applied such that compression is achieved at the supracondylar level for both columns. Plates must be strong enough and stiff enough to resist breaking or bending before union occurs at the supracondylar level.

The surgical approach did not correlate significantly with outcomes. Similarly, other evidence does not provide convincing support for one approach compared to another. Some data support superior clinical outcomes with an olecranon osteotomy, especially for comminuted C-type fractures compared to alternatives such as the triceps-sparing approach, while other data show no significant difference among approaches [1, 24–28].

Lastly, the surgeon must consider the ulnar nerve in treating distal humerus fractures. The orthopedic community does not have a consensus on whether to leave the nerve undissected, to release it *in situ*, or to transpose it. Our data do not support one method compared to another. Some evidence cautions against transposition [29]. A 2018 systematic review concluded that transposition does not have a protective effect against ulnar neuropathy after surgical repair of distal humerus fracture [30].

Distal humerus fractures are serious injuries with a high complication rate. In our case series, the use of fewer than three articular screws for AO C-type fractures and male gender were significant risk factors for complications including elbow stiffness and revision surgery. Plate configuration and surgical approach were not associated with an increased risk of complication.

## Conflict of interest

Charles M. Jobin, MD: Dr. Jobin receives consulting payments from Acumed, Depuy/Synthes, Intergrated Shoulder Collaboration, Intergra, Wright-Tornier, and Zimmer-Biomet, which are not directly related to the subject of this work. Dr. Jobin received grant support from American Shoulder & Elbow Surgeons related to the subject of this work and Grant funding from Orthopedic Scientific Research Foundation related to this research. Dr. Jobin is on the editorial board of Journal of American Academy of Orthopedic Surgeons (JAAOS). William N. Levine, MD; David Kovacevic, MD; Brian F. Grogan, MD; Nicholas C. Danford, MD; Stephen P. Maier II, MD; Pinkawas Kongmalai, MD; and Cesar D. Lopez, BS: The author(s), their immediate family, and any research foundation with which they are affiliated have not received any financial payments or other benefits from any commercial entity related to the subject of this article
